# Antimicrobial Resistance Development Pathways in Surface Waters and Public Health Implications

**DOI:** 10.3390/antibiotics11060821

**Published:** 2022-06-18

**Authors:** Joseph Kusi, Catherine Oluwalopeye Ojewole, Akinloye Emmanuel Ojewole, Isaac Nwi-Mozu

**Affiliations:** 1 Department of Environmental Sciences, Southern Illinois University Edwardsville, 44 Circle Drive, Campus Box 1099, Edwardsville, IL 62026, USA; cojewol@siue.edu (C.O.O.); aojewol@siue.edu (A.E.O.); 2 Schmid College of Science and Technology, Chapman University, One University Drive, Orange, CA 92866, USA; nwimozu@chapman.edu

**Keywords:** antibiotics, aquatic systems, bacteria, resistant pathogens, phenotypes, resistance genes

## Abstract

Human health is threatened by antibiotic-resistant bacteria and their related infections, which cause thousands of human deaths every year worldwide. Surface waters are vulnerable to human activities and natural processes that facilitate the emergence and spread of antibiotic-resistant bacteria in the environment. This study evaluated the pathways and drivers of antimicrobial resistance (AR) in surface waters. We analyzed antibiotic resistance healthcare-associated infection (HAI) data reported to the CDC’s National Healthcare Safety Network to determine the number of antimicrobial-resistant pathogens and their isolates detected in healthcare facilities. Ten pathogens and their isolates associated with HAIs tested resistant to the selected antibiotics, indicating the role of healthcare facilities in antimicrobial resistance in the environment. The analyzed data and literature research revealed that healthcare facilities, wastewater, agricultural settings, food, and wildlife populations serve as the major vehicles for AR in surface waters. Antibiotic residues, heavy metals, natural processes, and climate change were identified as the drivers of antimicrobial resistance in the aquatic environment. Food and animal handlers have a higher risk of exposure to resistant pathogens through ingestion and direct contact compared with the general population. The AR threat to public health may grow as pathogens in aquatic systems adjust to antibiotic residues, contaminants, and climate change effects. The unnecessary use of antibiotics increases the risk of AR, and the public should be encouraged to practice antibiotic stewardship to decrease the risk.

## 1. Introduction

Antimicrobial resistance (AR) in the environment is one of the emerging threats to public health globally. The total number of annual deaths caused by antimicrobial-resistant pathogen-related infections is estimated to increase from 700,000 to 10 million by 2050 with a USD 100 trillion cost worldwide [1]. In the United States alone, antibiotic-resistant pathogens cause about 2.8 million illnesses and 35,000 deaths annually [2,3,4]. According to the World Health Organization (WHO), the impact of AR on humans goes beyond health. The WHO estimates that AR could increase the number of poor people by 28 million by 2030 in low- and middle-income countries due to health disparities and the high cost of treating resistant infections [5]. The Impacts of AR on human health will continue to rise due to the increasing use of antibiotics in hospitals, nursing homes, and agricultural settings.

Antimicrobial resistance in the environment has been linked to the over-prescription of antibiotics, drug overdose, and improper disposal of antibiotic residues [6]. Prescriptions of antibiotics for outpatients are extremely high in the United States. In 2015 for instance, 269 million antibiotics were dispensed from outpatient pharmacies and more than 30% were prescribed incorrectly [3]. The frequent use of antibiotics in developing countries to treat infectious diseases due to inadequate water, sanitation, and hygiene (WASH) also contribute to the development of AR [5]. In developed countries, the widespread use of antibiotics in hospitals to treat infectious diseases or to mitigate infections during surgeries, hygiene, and sanitation may lead to AR [7]. Some of the dispensed antibiotics may end up in sewage and wastewater at homes and healthcare centers.

Antibiotics in wastewater are not completely removed by treatment processes, contributing to antibiotics in aquatic systems [8,9,10]. Several antibiotics persist in surface water and their measured concentrations range from 0.001 to 484 µg/L worldwide [11]. Studies have shown that the introduction of antibiotics in aquatic systems is associated with increasing numbers of antimicrobial-resistant bacteria (ARB) and emerging resistance genes [12,13]. Sub-inhibitory concentrations of antibiotics in aquatic systems influence horizontal gene transfer (HGT) and mutagenesis in bacteria [13]. As a result, several pathogens have acquired resistance to the most effective antibiotics and the faster rate at which microbes develop resistance to new antibiotics is not clearly understood. Microorganisms that develop resistance spread the resistance genes in the environment and pass them on to the next generation [14].

**Figure 1:**
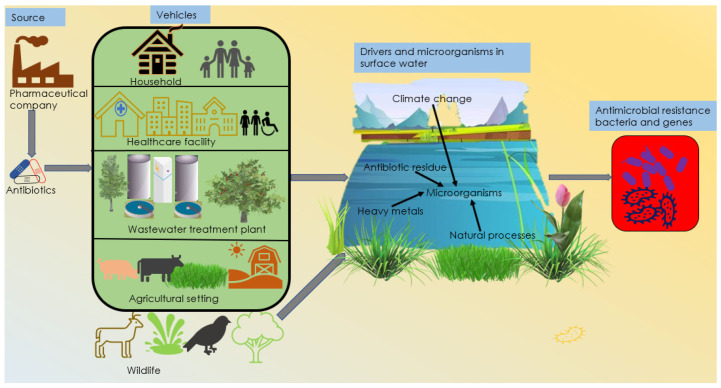
Antimicrobial resistance development pathway model showing the sources, vehicles, and drivers of antimicrobial resistance in the aquatic environment.

Surface waters have been identified as potential reservoirs for antibiotics and antibiotic resistance [15,16]. Many studies have detected antibiotic resistance pathogens and genes in lakes, rivers, streams, ponds, and estuaries in several countries (Table 1). Most studies in Table 1 focused on the detection of resistance genes, and their sources remain unknown. Some of the surface waters that harbor antimicrobial resistance genes (ARGs) are used for recreational activities, which present a potential risk of human exposure to resistant pathogens [17,18]. Primary sources and the development of AR in surface waters are not completely understood, which suggests the need for an investigation into the sources of AR in aquatic systems. Antibiotic resistance occurs when microorganisms do not respond to antimicrobial drugs designed to kill them [19]. The mechanism of developing AR in aquatic systems involves several factors. The combination of vehicles and drivers of AR enhances the development and spread of resistance in the environment. Healthcare facilities, wastewater, agricultural settings, food, and wildlife population are the major vehicles, while antibiotic residues, heavy metals, natural processes, and climate change are the drivers of AR in surface waters (Figure 1). Microbial responses to biocides and synthetic antibiotics can lead to the development of resistance [20]. For example, an *Enterococcus faecalis* strain secreted bacteriocins to outcompete other bacteria and produced immunity proteins to prevent the self-killing and antimicrobial activity of microcins secreted by other bacteria [21].

While surface waters reserve and disseminate ARB in the environment, processes involved in the occurrence and development of resistance remain complicated. The pathways that lead to AR in surface waters are associated with human exposure to resistant pathogens via the fecal-oral route. The purpose of the current study was to identify the major components of AR pathways in surface waters and their health implications using peer-reviewed articles. The findings of this study can be used by researchers and environmental health professionals to develop interventions to address the rapid spreading of AR in healthcare facilities and surface waters to protect public health.

## 2. Primary Source of Antibiotics

Over the years, the increasing need for life-saving antibiotics has resulted in the large-scale use of varieties of pharmaceutically active compounds (PhACs) to produce drugs across the world. Consequently, the increase in antibiotic production has been accompanied by an increase in the number of pharmaceutical industry effluents, many of which are generated from both active pharmaceutical ingredient (API) manufacturing units and finished pharmaceutical products (FPP), which contain antibiotic residues in significant amounts [52]. Recent studies have identified pharmaceutical industry sites as the primary source of antibiotics and hotspots for the spread of AR in the environment and among humans and animals due to the presence of high concentrations of ARGs from pharmaceutical effluents [53,54]. Analyzed effluent from treatment plants that received wastewater from pharmaceutical industries showed higher concentrations of antibiotics than the effluent from treatment plants supplied with municipal wastewater [53]. PhACs in treatment plant effluent increased AR, ARGs, bacterial abundance, and water pollution in Asia, Europe, and North America [53,55,56,57,58]. Antibiotics and their compounds produced by the pharmaceutical industry are distributed in different environmental settings susceptible to AR development.

## 3. Vehicles/Pathways for Antimicrobial Resistance

Residues of antibiotics are released into the environment through manufacturing processes, human feces/urine and animal dung, agricultural activities, and food processing. Healthcare facilities, wastewater, agricultural settings, food, and wildlife serve as reservoirs and vehicles for transporting antibiotic residues and antimicrobial resistant-bacteria (ARB) within the environmental compartments (Figure 1). Humans and animals infected with ARB may spread resistance in the environment through wastewater and direct deposition of feces into surface waters. The fate and transport of antibiotic residues and resistant bacteria are influenced by the environment [54]. For instance, readily water-soluble antibiotics may dissolve in wastewater or runoff water, which may weaken their ability to cause AR before entering surface water. Some ARB are susceptible to extreme environmental conditions that reduce their chances of survival in the environment. Antibiotic residues and resistant bacteria that are not readily affected by adverse conditions may persist during transportation and enhance the development and spread of AR in surface water.

### 3.1. Healthcare Facilities

**Figure 2:**
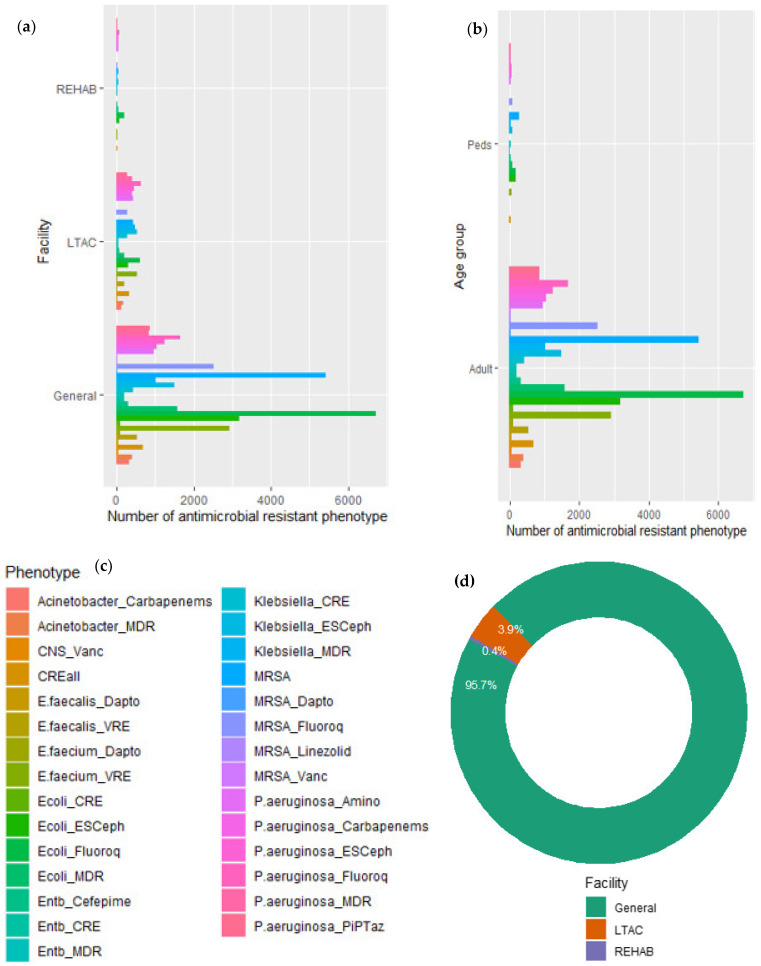
Antibiotic resistance phenotypes in healthcare facilities and patients. (**a**) Number of resistance phenotypes in healthcare facilities; (**b**) number of resistance phenotypes among age groups; (**c**) legend for (**a**,**b**); (**d**) proportion of resistance phenotypes among healthcare facilities. Peds = pediatrics; LTAC = long-term acute care; and REHAB = inpatient rehabilitation centers. See the full names of the phenotypes in Table 2. *n* (Adults) = 39,846,024, *n* (Peds) = 1,888,388.

The risk of patients and healthcare workers contracting antibiotic resistance healthcare-associated infections (HAIs) in healthcare facilities and the spread of resistant pathogens in the environment pose a severe threat to global health. Antibiotic resistance HAI data reported to the National Healthcare Safety Network (NHSN) at the Centers for Disease Control and Prevention (CDC) from 2011 to 2019 by general hospitals, long-term acute care hospitals (LTAC), and inpatient rehabilitation center (REHAB) facilities [59] were analyzed using RStudio software 4.1.2. (RStudio, Boston, MA, USA) version to describe the patterns for AR in healthcare facilities. Antimicrobial-resistant pathogens were distributed across the three healthcare facilities. Patients developed antibiotic resistance HAIs within 48 h after going through clinical procedures in healthcare facilities in the United States (Figure 2).

Clinical microbiology laboratories that reported pathogen and antimicrobial susceptibility data to NHSN identified a total of 29 phenotypes of ten pathogens that were resistant to a selected group of antibiotics (Table 2). Antimicrobial resistance phenotypes detected in patients varied among healthcare facilities. The highest number of phenotypes that showed resistance to the selected antimicrobials occurred in general hospitals (Figure 2a). *Escherichia coli* and *Staphylococcus aureus* isolates caused the highest number of antibiotic resistance HAIs in general hospitals while *E. coli*, *S. aureus*, *Pseudomonas aeruginosa*, and *Enterococcus faecium* isolates caused most HAIs in LTAC. In REHAB centers, HAIs were mainly caused by *E. coli*. The number of adult patients who developed antibiotic resistance infections was far higher than that of pediatrics (Figure 2b). Adults were infected with all identified pathogen isolates, but HAIs in pediatrics were mainly caused by *E. coli*, *Klebsiella, S. aureus,* and *P. aeruginosa* isolates (Figure 2b). Adults tend to take more antibiotics and visit LTAC and REHAB more than children, which may partly account for their higher total number of resistant phenotypes compared to that of pediatrics. Approximately 96% of the total number of resistant phenotypes was in general hospitals, 4% was detected in LTAC, and less than 0.5% was detected in REHAB (Figure 2d).

Physicians prescribe antibiotics for inpatients to treat bacterial infections, so pathogens may be continuously exposed to antibiotics at sub-inhibitory concentrations. Exposure to sub-minimal inhibitory concentrations of antibiotics causes genetic mutations and persistence that affect bacterial phenotypes [60]. The effects of sub-minimal inhibitory concentrations of antibiotics may have contributed to the emergence of the various pathogenic resistant phenotypes detected in the healthcare facilities, which serve as a vehicle for the dissemination of antibiotic-resistant pathogens in the environment (Figure 1). Patients with HAIs in general hospitals, LTAC, or REHAB centers can transmit antibiotic-resistant pathogens to other patients and health workers within the healthcare facilities. Hospital-acquired infections may turn into community-acquired infections when discharged patients with untreated or undetected HAIs spread antimicrobial-resistant pathogens in their communities. In addition, infected patients may shed antibiotic-resistant pathogens in their feces and urine which may introduce AR into wastewater. Previous studies detected various ARGs in hospital wastewater [12,61], indicating a potential transmission of AR from healthcare facilities to the aquatic environment.

### 3.2. Wastewater

Large volumes of wastewaters released from households, schools, businesses, industries, healthcare facilities, and agricultural settings eventually find their way into watercourses. Antibiotics consumed by humans are released into sewage and septic tanks which harbor a variety of microbial populations. A large proportion of active ingredients in antibiotics enter the environment via human and animal wastes [60]. Metabolites of consumed drugs, antibiotics, and stimulants excreted in urine and feces from households contribute to antibiotic compounds in municipal wastewater systems [62]. Antibiotics consumed by humans undergo a series of transformations to form antibiotic compounds or metabolites before they are excreted from the body. The metabolites are used as biomarkers to identify and estimate the concentrations of specific antibiotics in wastewater. A group of researchers detected the metabolites of macrolides (N-RTM and Des-ATM) and sulfonamides (N-SPY and N-SMX) in wastewater with their concentrations ranging from 1.2 to 772.2 ng/L [63], demonstrating the presence of antibiotic compounds in municipal wastewater. The release of antibiotic metabolites into wastewater is worsened by the rising self-medication practices across the world where up to 50% of over-the-counter medicines are antibiotics [64,65]. The inappropriate use and distribution of antibiotics in households have been linked to the development of AR [66,67].

Globally, many healthcare facilities discharge raw wastewater, potentially containing antibiotic residues and antibiotic-resistant pathogens, into municipal sewer systems [12]. Similarly, wastewater from pharmaceutical companies contains abundant ARGs [42]. The occurrence and concentration of ARGs are influenced by wastewater and water chemistry [32]. Evidence from previous studies shows that wastewater from pharmaceutical companies and slaughterhouses contributes to the diversity of antibiotic resistance and pathogens in surface waters [42]. High levels of multi-drug resistance (MDR) bacteria including *E. coli*, *Klebsiella pneumoniae*, and *Enterobacteriaceae* were detected in wastewater from pig and poultry slaughterhouses [10,68,69,70], demonstrating the contribution of abattoirs to the spread of AR in aquatic systems and pathogen exposure risks posed to slaughterhouse employees. In [42], a similar diversity of pathogens among river water and slaughterhouses was found. Non-point sources of pollution such as stormwater have also been found to contribute to antibiotic resistance in aquatic systems [39].

#### 3.2.1. Microorganisms in Wastewater

Some groups of microorganisms in wastewater, such as enteric bacteria, viruses, and protozoan cysts, pose threats to human health [71], while other groups are useful in wastewater treatment. Bacteria are used in wastewater treatment facilities to break down organic matter and chemical pollutants in wastewater. Microbial composition and function are important factors that influence the efficiency of wastewater treatment. The presence of different microbial populations can increase the competition for food and space among microorganisms. Some microbes release toxins to prevent the growth of other microbes, which can lead to the development of AR in bacteria. Microbes are also exposed to antibiotics in wastewater as they feed on organic matter. Antibiotics such as oxytetracycline, tetracycline, sulfadiazine, and sulfamethoxazole have been detected in wastewater [9]. These antibiotics persist in the aquatic environment and are very effective in stimulating resistance even at lower concentrations [4,13], probably because they are in soluble form. Laboratory experiments have confirmed the resistance of fecal indicator bacteria to many antibiotics in wastewater [72], but the mechanism of developing resistance to antibiotics is unclear.

Before 1950, natural products with antibiotic activity were used to treat microbial infections with no evidence of emerging AR. A paradigm shift occurred in the 1950s when pharmaceutical companies produced antibiotics containing synthetic derivatives for human use [73,74]. Since then, large quantities of antibiotics have been manufactured and released into the environment through their applications for treating microbial diseases. The continuous applications of antibiotics caused microbes to develop tolerance, leading to the development of antibiotic resistance [75]. A study found a correlation between antibiotic concentrations and an increasing number of ARB in wastewater and went further to quantify the number of ARB in antibiotic-loaded wastewater and compared them with the numbers in raw wastewater [76]. The number of resistant bacteria was always higher in the antibiotic-loaded wastewater, suggesting an association between antibiotic exposure and ARB. The potential development of resistance in wastewater underscores its important role in the transmission of resistant pathogens in surface water.

#### 3.2.2. Controversy over Wastewater Treatment Process

The main goal of wastewater treatment is to remove pollutants including microorganisms before discharging the treated water into surface water bodies to protect public health and ecosystem health. Wastewater treatment facilities apply the available technologies to remove pathogens from wastewater [8], but resistant pathogens have been detected in treated wastewater, raising concerns about the efficiency of wastewater treatment technologies [77]. For example, multidrug-resistant *E. coli*, *Enterococcus faecalis*, and *Enterococcus faecium* were detected in treated wastewater [9,78]. Another study showed that the prevalence of antibiotic resistance increased from raw effluent to final effluent and was higher downstream of the receiving waterbody [79]. The release of treated wastewater containing multidrug-resistant bacteria into watercourses may spread AR in surface waters.

The development of antibiotic resistance in the effluent may be part of the reasons why chlorination has not been effective in killing pathogens during wastewater treatment. In the United States, some wastewater treatment facilities use iodination, ozonation, and ultraviolet (UV) radiation to improve the removal of pathogens [8]. Advanced and efficient wastewater treatment methods are needed to remove pathogens from wastewater. The effectiveness of the disinfection of wastewater may depend on the type of method and conditions. Survival studies of pathogens in wastewater may help to compare the effectiveness of disinfection methods. A membrane biological reactor (MBR) is more effective for killing resistant pathogens in sewage compared to chlorination and UV methods while biosolid treatment methods such as anaerobic digestion and lime stabilization eliminate more pathogens compared to dewatering and gravity thickening [9]. The effectiveness of UV depends on wastewater chemistry, the intensity of UV light, and the exposure time of the microbes. UV kills most viruses, bacteria, spores, and cysts. UV has no residual effects but becomes ineffective at low dosages and high turbidity [71].

A comparison of methods may reveal inefficient disinfection strategies that can contribute to the transfer of resistant pathogens from treated wastewater to surface waters used as sources of drinking water. The release of pathogens into surface water can also serve as a fecal-oral route for human exposure to pathogens, which is a major means of waterborne disease transmission. Since AR is present in wastewater, inefficient wastewater treatment systems can lead to AR in surface waters and adverse health outcomes. None of the available disinfection methods is 100% efficient in eliminating pathogens, supporting the argument that the inefficiency of wastewater treatment technologies can contribute to the development and dissemination of antimicrobial-resistant pathogens in aquatic systems. Although treatment technologies may contribute to AR, they may not be the primary source of resistant bacteria in aquatic systems. Agricultural activities, wildlife populations, climate change, and natural processes may cause greater resistance in the aquatic environment.

### 3.3. Agricultural Settings

Farmers worldwide use antibiotics to treat infections in animals to increase farm yields. Unfortunately, some pathogens have developed resistance to different classes of antibiotics commonly used to treat animal diseases [80,81], raising concerns about their implications on human health. In developed countries, the excessive use of antibiotics in food-producing animals is among the main contributors to antibiotic resistance [7]. Antibiotics use and AR in farm animals impose economic costs and health burdens on humans. The external cost of administering 1kg of antibiotics (fluoroquinolones) in broiler chicken production was estimated to be USD 1500 [82], which affects farmers, farmworkers, and consumers. Antimicrobial resistance in farm animals presents occupational hazards to farmworkers and the risk of foodborne outbreaks in communities. Many foodborne outbreaks in the United States originate from the farms where animal or crop products are produced [3]. Farmers and food vendors incur financial losses when food items are recalled due to potential pathogen contamination. Regulations that target the use of antibiotics in animal farms also increase the cost of animal products borne by farmers and consumers [83]. As a result, low-income families may not be able to afford animal products to meet their nutritional needs, especially for children. Agricultural activities affect not only humans but aquatic systems as well.

Agriculture is one of the major human activities that impacts the aquatic ecology of microorganisms. The pressure on agriculture to feed the growing human population has increased the use of antibiotics to disinfect farm animals which may have contributed to the selection of more antibiotic-resistant pathogens [84]. Livestock and poultry farmers apply antibiotics to prevent or control microbial infections. Some animal farms, especially livestock, are sited near waterbodies to provide easy access to water sources for the animals. The direct deposit of animal wastes and runoff from such farms contaminate surface waters with antibiotic residues and fecal bacteria. For example, higher concentrations of sulfamethoxazole, sulfapyridine, trimethoprim, erythromycin-H_2_O, azithromycin, clarithromycin, and roxithromycin associated with livestock were detected in a river with catchment areas which were dominated by pasture grazing [13]. The presence of these antibiotics in surface water can stimulate AR.

Some pathogens may develop resistance in the intestines of farm animals and may be excreted with their wastes. Animal manure applied on soil for the cultivation of crops may disseminate ARB in the environment [85]. Runoff from farmlands may carry animal manure and ARB into surface waters used for irrigation, recreational, and drinking purposes. Furthermore, the processing of animal manure to generate biogas has been shown to increase the number of ARB and ARGs [80]. Additional treatment is needed to remove resistant pathogens from digested animal manure before applying it on farmlands to prevent the spread of AR in the environment.

### 3.4. Food

Food plays a significant role in the transmission of microorganisms and foodborne pathogens [86]. Food also acts as a vehicle for the transfer of ARB and ARGs to humans [87]. Excessive use of antibiotics occurs in the production of animal food (meat, eggs, and milk) and the application of food additives for colorization and preservation. The overuse of antibiotics in food has increased human exposure to AR [88]. The consumption of raw food, fresh vegetables, and fruits is another source of human exposure to antibiotic residues, ARGs, and ARB [89,90,91]. Contamination of many foods and farm produce with pathogenic bacteria may occur during production, transportation, irrigation, and handling of animal waste to produce manure and biosolids as fertilizers [92,93,94]. Some crop-producing farmers use surface waters for irrigation and may be exposed to antimicrobial-resistant pathogens. Irrigation water that contains antimicrobial-resistant pathogens can contaminate crops to cause foodborne outbreaks. There are instances in the United States where the CDC traced sources of foodborne illness outbreaks to contaminated irrigation water at farms using the food production chain method [3]. Farms that use water contaminated with pathogens for irrigation may spread foodborne pathogens and increase human exposure through the consumption of contaminated food crops.

Each year in the United States, the CDC estimates that 48 million people get sick from foodborne illnesses. About 742,000 cases of foodborne illnesses are caused by ARB [95]. For example, *Salmonella* species resistant to antibiotics in different types of food (raw chicken, eggs, and dairy products) have been reported in the United States by the CDC based on epidemiologic and laboratory evidence. In isolated samples collected from 139 food items and 97 infected people, all samples showed resistance to antibiotics such as chloramphenicol, tetracycline, ampicillin, nalidixic acid, streptomycin, ciprofloxacin, Fosfomycin, gentamicin, kanamycin, hygromycin, and trimethoprim-sulfamethoxazole [96].

The link between antimicrobial-resistant *Salmonella typhimurium* from pigs to humans has been reported (Table 3). *Salmonella typhimurium* isolated from pigs and humans has been found to develop AR to a wide range of antibiotics. *Salmonella typhimurium* DT104 exhibits the most prevalent resistance to ampicillin, chloramphenicol, streptomycin, sulfonamides, and tetracycline for pork isolates (7.4%) and human isolates (13.2%) [97]. A similar occurrence of *S. typhimurium* DT104 AR has been reported in milk/milk cheese [98,99], dried anchovy [100], lettuce [101], and ground beef [102].

Antimicrobial-resistant genes and antimicrobial residues can be transferred to humans through fish and prawns raised in aquaculture. Prawns and pangasius fillets imported to Demark from Asia harbored *E. coli* and produced blaCTX–M–15 and blaCTX–M–55 resistance genes against cephalosporins, macrolides, colistin, and fluoroquinolones [103]. In a similar study in Nile tilapia fillets cultured in Brazil, ref. [104] reported a high prevalence of *Salmonella* spp. and a high AR rate for amoxicillin/clavulanic acid (87.7%), tetracycline (82.5%), and sulfonamide (57.9%).

During food production, some bio-preserving microorganisms, starter cultures, bacteriophages, and probiotics that contain ARB are intentionally added for preservation to extend the shelf life of food [87]. Starter cultures are resistant to tetracycline. In fermented food and probiotic strains, enterococcus in dairy products, lactobacillus in cheese, and lactococcus are known to be resistant to vancomycin, tetracycline, erythromycin, and chloramphenicol [105,106]. In raw meat, lactic acid bacteria, have also been identified to be resistant to tetracycline [107]. *Staphylococcus* isolates obtained from starter cultures in meat and *Bifidobacterium lactis* have been found to be resistant to tetracycline while *Lactobacillus reuteri* showed resistance to lincosamide [108]. Coagulase-negative *Staphylococci* (CNS) strains associated with food and starter culture isolated from cheese (87%), sausage (83%), and meat starter culture (93%) were found to be resistant to chloramphenicol, clindamycin, cotrimoxazole, gentamicin, kanamycin, linezolid, neomycin, streptomycin, synercid, and vancomycin [109].

**Table 3:** Antimicrobial resistance in food and the most affected group of people.

Source	Exposure Route	Risk Group	Resistant Bacteria/Gene	Reference
Maize	Ingestion Direct contact	Poultry workers Market workers	*E. coli*	[68]
Chicken	Direct contact Ingestion	Farmworkers, slaughterhouse workers, veterinarians	Methicillin-resistant *S. aureus* (LA-MRSA). *E. coli*	[68,110] [69]
Vegetables, fruits, fish, and dairy products	Ingestion Direct contact	Long term storage consumers Farmers	*Sitotroga cerealla* *Salmonella* Campylobacter	[68,87,111,112]
Beef	Direct contact with livestock Fecal oral route	Agricultural workers Meat consumer	*S. typhimurium* DT104	[113]
Chicken, beef, pork	Ingestion Skin contact	General population	*Salmonella enterica*	[114]
Chicken, beef, fish	Direct contact	Veterinarians, Farm workers,	*E. coli* methicillin-resistant *S. aureus*	[115]
Chicken, turkey, bovine, porcine meat	Ingestion Direct contact	Food handler Health workers	*E. coli, S. typhimurium* *Klebsiella pneumoniae*	[115,116]
Beef, chicken, pork, lamb, duck, egg, milk, vegetables, seafood	Food contact surface Ingestion Direct contact	Farm workers Food service worker	*Leuconostoc pseudomesenteroides*, *Lactobacillus pentosus*, *Salmonella enteritidis*	[117,118,119]
Milk	Direct contact Ingestion	Poultry workers Market workers	*E. coli*, *S. aureus*	[68,112]

### 3.5. Wildlife Populations

Wild populations are reservoirs of microorganisms and can spread ARGs in the environment [120]. Mammals and birds are wild animals that harbor large amounts of ARGs in their guts [121]. Fecal samples collected from birds and mammalian species contained *E. coli* isolates that exhibited multidrug-resistant phenotypes after testing for their susceptibility to seven antimicrobial agents [122]. Similarly, about 90% of bacterial isolates from wild rodents were found resistant to beta-lactam antibiotics [123]. Wildlife carrying ARGs is concerning for public health and animal health. Resistance phenotypes may be transferred from wild birds and mammals to food-producing animals that share the same environment with the wildlife [122]. Wild animals may spread AR in surface waters through their feces and swimming [124]. Humans may be infected with zoonotic antimicrobial-resistant pathogens through their interactions with wild animals and their habitats or by killing the animals for food. For example, the deadliest epidemics and pandemics such as Ebola, Severe Acute Respiratory Syndrome (SARS), Middle East Respiratory Syndrome (MERS), and COVID-19 are believed to have originated from human interactions with wild animals [125,126]. The transfer of novel and resistant pathogens from wildlife populations to humans leading to fatal infectious diseases underscores the serious public health implications for our close relationship with wild animals.

Although wildlife populations contribute to the spread of novel pathogens and ARGs in the environment, this is not included in many statistical models for microbial studies due to limited information on the density distribution of common wildlife and the difficulty in the quantification of microorganisms released from their fecal loads [127]. Wildlife populations can be included in models such as the soil and water assessment tool (SWAT) as a nonpoint source to monitor the survival and transport of microorganisms released from wildlife into surface waters. The model can be used to determine the movement of AR between humans and wildlife populations.

Previous studies demonstrated a connection between human population density and wildlife AR [128,129,130]. Wild animals in highly human-populated areas had greater AR compared to animals located in less dense human-populated areas [123], suggesting a human influence on AR in wild animals. The results of these studies imply that there can be an exchange of resistance genes between humans and wildlife which may occur through surface waters. In contrast, there are isolated places with no human activity where AR has been detected, raising questions about the mechanism of resistance development and ancestral genes [9,121,131,132].

The mechanism of developing resistance in human-impacted areas and pristine environments may differ due to the difference in ecological landscapes. Antimicrobial resistance in places with no history of anthropogenic activities and antibiotic exposure may have evolved through microbial responses to toxins and extreme environmental conditions and/or through the transfer of ancestral resistance genes from one generation to another. There are several historical genes and antibiotic-producing species causing resistance to natural antibiotics in the environment and may serve as the origin of resistance genes in pristine environments [132]. Resistance genes available in the environment may be acquired by organisms to develop AR. Microorganisms may produce strains resistant to variations in environmental conditions or suitable for adaptation to adverse conditions. It is also important to understand that some wild animals with acquired resistance genes can migrate from human-impacted areas to pristine environments and spread the resistance genes. MDR *E. coli* isolates were detected in migratory birds in both northern and southern China, but the rate of resistance was higher in the south [124], which confirms the role of the environment in the spread of AR. Animal migration may interfere with our understanding of the origin of AR in pristine environments because the sources of resistant bacteria carried by migratory animals are usually unknown.

## 4. Drivers of Antimicrobial Resistance

There are many factors, including natural processes, water chemistry, antibiotic residues, biocides, heavy metals, and climate change, that drive the mechanism of antimicrobial resistance in surface water [20]. These drivers influence bacteria in surface water to develop AR (Figure 1). Some of the drivers are already present in the water or may be transported to the waterbodies through the vehicles of AR. The interactions between the drivers or between the drivers and vehicles may promote AR in surface waters. For example, climate change and natural selection may favor the growth and survival of a particular ARB in the aquatic environment.

### 4.1. Natural Processes

Antimicrobial resistance in the environment can occur naturally by mutagenesis and the acquisition of resistant genes through horizontal gene transfer [120]. Several resistant genes can be mobilized in transposons, integrons, or plasmids and transferred to other bacteria [75,133]. Microorganisms, plants, and animals have been producing natural antibiotics and antimicrobial compounds for many years for self-defense and competition for resources [60]. Antibiotics and their compounds produced by living organisms may stimulate mechanisms for AR development. Generally, microorganisms respond to antimicrobial compounds and toxins in the environment by producing mechanisms that can inactivate or destroy the active agents in toxic compounds. Bacteria may develop AR through the formation of impermeable barriers, multidrug resistance efflux pumps, mutations, inactivation of antibiotics, and exchange of genetic information [133]. Some of these mechanisms can cause gene mutation in microorganisms which may result in permanent DNA alterations leading to the development of resistance to similar compounds or toxins in the environment. Antimicrobial resistance spreads in the environment through physical forces such as water and wind which serve as reservoirs for resistance genes.

The aquatic environment harbors diverse microbial communities, increasing competition within and between species for resources and space. During competition, some microbes secrete toxins to limit the growth of others and increase their chances of survival. Other microbes may respond by producing resistance genes responsible for the modification or degradation of the toxins. Limited resources may increase competition and the production of resistance to enhance survival [21,134]. The changes in environmental conditions and human activities increase selection pressure for the emergence and persistence of AR [131]. Patterns of geographic variation have been linked to the resistance to antibiotics, suggesting the role of environmental factors in selection pressure for resistance [6,135]. Factors influencing geographic variations, in turn, affect selection pressure. Physical, chemical, and biological forces in the environment also apply additional selective pressure on existing ARGs. For example, extreme temperatures, wind, and heavy rainfall drive the spread of resistance genes in the environment [20,120].

Nature is not the only selective pressure for antibiotic resistance. Anthropogenic activities play an important role in selection pressure. Antimicrobial resistance was found to be highest in urban areas compared to rural areas [22]. Antibiotic resistance also varies from country to country due to the large quantities of antibiotics consumed by individuals, indicating the selective pressure that antibiotic use exerts on resistance. People in developed countries consume large volumes of antibiotics because their healthcare policies and socioeconomic factors make antibiotics easily accessible compared to developing countries [20]. The control of antibiotic prescription in some countries has shown a decrease in antibiotic resistance [6]. The association between antibiotic use and antibiotic resistance found in previous studies demonstrates how human activities influence the selection pressure for resistance. Human activities impose selective pressure on resistant bacteria which increases the evolution and spread of resistance genes in the environment [123]. As a result, microorganisms may produce phenotypes suitable for the environment to enhance adaptation.

### 4.2. Heavy Metals

Heavy metals are vastly distributed in many water systems and constitute a major component of the anthropogenic household, agricultural, and industrial waste disposal sites [136]. At low concentrations, heavy metals are toxic and constitute a threat to public health [137,138]. Heavy metals can remain in the environment for a while and pose a long-term selective pressure on the maintenance and proliferation of antibiotic resistance [139]. In natural environments, sites contaminated with heavy metals have been found to contain high levels of antibiotic resistance microorganisms [140,141]. Bacteria that react to the inducement of metals through the formation of heavy metal resistance genes (MRGs) and ARGs have also been discovered in heavy metal-contaminated sites [142].

Evidence from previous studies has shown the influence of heavy metals on the resistance of bacteria to antibiotics, resulting in the discharge of ARGs into the environment and paving the way for human hosts [143]. Heavy metals like copper, zinc, nickel, arsenic, cadmium, and mercury have been reported to provide a co-selection pressure for antibiotic resistance of proteobacteria and actinobacteria [144,145,146,147]. The co-selection mechanism mainly involves co-regulation, co-resistance, and cross-resistance [148]. When antibiotics and heavy metals exist in the environment, bacteria may form resistance through the co-resistance mechanism, while through cross-resistance mechanisms, bacteria can activate the efflux pump protein and thus become resistant to heavy metals and antibiotics. The co-regulation mechanisms occur when bacteria are subjected to the stress of antibiotics and heavy metals. This condition makes the bacteria respond to the signal transduction system such as the two-component system thus making the bacteria resistant to heavy metals [149].

Mining activities are known to generate a variety of heavy metals. In [150], the pollution of zinc, nickel, and manganese and the characteristics of ARGs in mining-affected waters in China were investigated. The abundance of ARGs, with a great proportion of chloramphenicol, sulfonamides, and tetracycline resistance genes, showed a significant correlation with the concentrations of heavy metals found in mining water when compared with those without mining activities [150]. In mining waters, a high microbial composition and diversity were observed where bacteroidetes, proteobacteria, and actinobacteria were the most prevalent hosts for ARGs. In a similar study, ref. [142] found arsC and ereA genes coding for resistance mechanisms to arsenic and cadmium in heavy metal-polluted copper tailings dam areas in northern China.

Wastewater treatment plants (WWTPs) have been identified as an important hotspot for the evolution and dissemination of AR [76,151]. Activated sludges contain significant amounts of heavy metals, thus creating suitable conditions for microorganisms to co-select and spread AR [152]. In urban wastewater in Shanghai, China, ref. [139] observed that zinc, lead, copper, cadmium, nickel, and chromium imposed significant selections on the proliferation and dissemination of erythromycin resistance genes. The findings of [153] indicated that the presence of relatively low heavy metal levels (arsenate 2 mM, copper 4 mM, or zinc 1.25 mM) in polluted environments can enhance bacterial antibiotic resistance in bacterium LSJC7 and *E. coli* DH5α.

Metal-based nano-enabled materials with antimicrobial properties such as silver nanoparticles (AgNPs) are incorporated in antibiotics and consumer products to kill or prevent the growth of bacteria. AgNPs are highly toxic to bacteria and are considered promising antimicrobial agents to address the growing threats posed to public health by AR. For example, AgNPs caused a rapid decline in survival and metabolic activity of microorganisms in surface water [154,155]. Additionally, AgNPs were effective in disrupting the morphology and structure and inhibiting biofilm formation of multidrug-resistant *Pseudomonas aeruginosa* [156,157]. In contrast, recent studies have shown the potential of AgNPs to stimulate AR in *Escherichia coli* K-12 MG1655 strain and the transfer of ARGs from *E. coli* K-12 LE392 to *Pseudomonas putida* KT2440 at environmentally relevant concentrations [158,159]. The results of these studies demonstrate that microorganisms are gradually becoming resistant to AgNPs as large quantities of the nanoparticles are produced annually and incorporated into consumer products worldwide. Bacterial resistance to AgNPs may be partly due to the known characteristics of metal-inducing antimicrobial resistance in bacteria [158]. Metal-tolerant bacterial communities were found to be resistant to tetracycline and vancomycin [141]. This suggests that bacteria that are resistant to AgNPs may exhibit similar resistance to available antibiotics. The development of AR to AgNPs poses a threat to public health because the promising antimicrobial agent could no longer kill microbes or inhibit the growth of pathogenic bacteria in many consumer products.

### 4.3. Climate Change

Climate change increases temperature, rainfall, runoff, and drought [160]. Waterborne and vector-borne disease outbreaks that follow flooding have been reported worldwide [161,162]. Heavy rains transmit and spread pathogens through run-offs in surface waters [160,163]. Climate change alters human and animal shelters, resulting in overcrowding and the spread of diseases. Extreme drought increases water scarcity and reduces sanitation [164], compelling people to use water sources of poor quality for drinking, domestic purposes, and irrigation, which may increase the risk of human exposure to pathogens.

The association between climate change and the development of AR is gradually gaining attention due to the influence of extreme weather conditions on the growth and activity of pathogens. The expression and transmission of genes responsible for phenotypic resistance may be triggered by environmental stressors [120]. Antimicrobial resistance in common pathogens increases with increasing temperature. In [165], an association between climate change and AR in the United States was demonstrated. The group found that an increase in temperature of 10 °C correlated with an increase in AR pathogenic bacterial species (*Escherichia coli*, *Klebsiella pneumoniae*, and *Staphylococcus aureus*). Although the association between temperature increase and pathogens does not mean climate change causes an increase in AR, it suggests an increasing selection pressure on antibiotic-resistant pathogens at higher temperatures. Increasing temperatures may facilitate the development and dissemination of antibiotic-resistant pathogens, which may worsen the existing public health issues related to AR.

Climate change influences the fate and transport of antibiotic-resistant pathogens in the environment [84,164]. Changes in climatic conditions affect biological and ecological processes that influence the transmission of pathogens and infectious diseases. Pathogenic microorganisms have low homeostasis and lack thermostatic mechanisms; therefore, their metabolic activity, temperature, and fluid levels are controlled by the local climate [166,167]. These organisms have limits of temperature variation that they can tolerate, and beyond which they may stop reproduction or die [167]. The lack of a thermostatic mechanism can cause pathogens to develop resistant genes that can withstand adverse environmental conditions and cause human diseases.

Water chemistry, water volume, and water flow rate are affected by climate change, which in turn influences the antibiotic properties and metabolic activity of pathogens. The persistence of antibiotics in the environment depends on water chemistry and fate processes [84]. High pH and low organic matter content can increase the dissolution and bioavailability of antibiotics in aquatic systems. Antibiotics may undergo transformation, biodegradation, and photooxidation to reduce their toxicity, but some antibiotics persist in the environment and exert their toxic effects on microorganisms. Low water volume and flow rate as a result of drought can increase concentrations of antibiotics and other contaminants in aquatic systems [163]. Pathogens respond to high antibiotic concentrations from effluent discharges by producing resistant genes to cope with the high levels of contaminants. Our knowledge of the role of climate change in AR is limited due to the lack of consistent data on climate variables, including temperature, precipitation, humidity, wind, and solar radiation.

## 5. Conclusions

Antimicrobial resistance is common in aquatic systems due to the presence of multiple determinants. Healthcare facilities, wastewater, agricultural settings, food, and wildlife serve as the major vehicles or pathways, while antibiotic residues, heavy metals, natural processes, and climate change serve as the drivers of antimicrobial resistance in surface waters. This study reported that healthcare workers, farmers, farmworkers, market workers, food service workers, slaughterhouse workers, and veterinarians have a higher risk of exposure to resistant pathogens compared to the general population due to the nature of their occupation (Table 3). Adhering to personal hygiene, safety, and good agricultural practices may help to reduce food contamination and foodborne illnesses. Humans may be exposed to waterborne pathogens in drinking water due to the failure of water treatment processes to eliminate resistant pathogens. The consumption of untreated water by people in communities that lack access to clean water may also contribute to waterborne illnesses. The current drinking water treatment and monitoring methods reduce human exposure to pathogens but communities that lack treated drinking water have greater risks of pathogen exposure. Several communities in developing countries lack access to potable water [168] and they use surface water as a primary source of drinking water and other domestic purposes, which may increase their risk of exposure to antibiotic-resistant pathogens. Ingesting ARB has the potential to cause medical treatment failures and limit the choice of antibiotics used in treatments. The continuous treatment of patients with antibiotics may result in gastrointestinal resistant pathogens occupying advantageous positions in the human body. Individuals exposed to resistant pathogens may suffer from incurable diseases or eventually die because there may be no antibiotics that can kill the emerging pathogens. The unnecessary use of antibiotics increases the risk of AR, and the public should be encouraged to practice antibiotic stewardship to decrease the risk.

## Figures and Tables

**Table 1:** Antimicrobial resistance detected in surface waters in different countries. ARGs = antibiotic resistance genes; MDR = multi-drug resistance; MRSA = methicillin-resistant *Staphylococcus aureus*.

Surface Water	Resistant Pathogen/Gene	Country	Reference
River watershed	Shiga toxin-producing *Escherichia coli*	Canada	[15]
Lake	Enterobacteriaceae	Brazil	[16]
Pond	ARGs	Bangladesh	[22]
Lake and river	ARGs	China	[23,24]
Lake	ARGs	China	[25,26,27,28,29,30]
River	*Escherichia coli* and *Klebsiella pneumoniae*	Lebanon	[31]
River	ARGs	Germany	[32]
River/sediment	ARGs	China	[33,34,35,36]
River	ARGs	Brazil	[37,38]
Stormwater	ARGs	United States	[39]
River	ARGs	Sri Lanka	[40]
River	ARGs	China	[36,41,42]
River	ARGs and MDR	India	[43]
River	ARGs	Germany	[44]
Lake	*E. coli*, ARGs, and MDR	Sri Lanka	[45]
Marine /lake/river	ARGs	Puerto Rico	[17]
River	ARGs	South Africa	[46]
River	*E. coli* and MDR	India	[47]
Estuarine	ARGs	Portugal	[48]
Lake/river	*Enterococcus faecalis*, *Enterococcus faecium*, *Enterococcus mundtii*, ARGs	Serbia	[49]
Lake/river/sediment	MDR	Germany	[50]
River	ARGs	Australia and Germany	[51]
Lake/river/stream	ARGs and MRSA	Portugal	[18]

**Table 2:** Pathogens and their phenotypes detected in patients with antibiotic resistance healthcare-associated infections (HAIs) in healthcare facilities. This table was adapted from NHSN annual reports, 2011–2019.

Pathogen	Phenotype	Abbreviation	Selected Group of Antimicrobials
*Escherichia coli*	Carbapenem-resistant (CRE)	*Ecoli*_CRE	Imipenem, meropenem, doripenem, ertapenem
	Cephalosporin-resistant	*Ecoli*_ESCeph	Ceftriaxone, ceftazidime, cefepime, cefotaxime
	Fluoroquinolone-resistant	*Ecoli*_Fluoroq	Ciprofloxacin, levofloxacin, moxifloxacin
	Multidrug-resistant (MDR)	*Ecoli*_MDR	Cephalosporins, fluoroquinolones, aminoglycosides, piperacillin/tazobactam
*Enterobacter*	Carbapenem-resistant (CRE)	*Entb*_CRE	Imipenem, meropenem, doripenem, ertapenem
	Cefepime-resistant	*Entb*_Cefepime	Cefepime
	Multidrug-resistant (MDR)	*Entb*_MDR	Cefepime, fluoroquinolones, aminoglycosides, piperacillin/tazobactam
*Klebsiella*	Carbapenem-resistant (CRE)	*Klebsiella*_CRE	Imipenem, meropenem, doripenem, ertapenem
	Cephalosporin-resistant	*Klebsiella*_ESCeph	Ceftriaxone, ceftazidime, cefepime, cefotaxim
	Multidrug-resistant (MDR)	*Klebsiella*_MDR	Cephalosporins, fluoroquinolones, aminoglycosides, piperacillin/tazobactam
*Pseudomonas aeruginosa*	Carbapenem-resistant	*P. aeruginosa*_Carbapenems	Imipenem, meropenem, doripenem
	Cephalosporin-resistant	*P. aeruginosa*_ESCeph	Ceftazidime, cefepime
	Fluoroquinolone-resistant	*P. aeruginosa*_Fluoroq	Ciprofloxacin, levofloxacin
	Aminoglycoside-resistant	*P. aeruginosa*_Amino	amikacin, gentamicin, tobramycin
	Piperacillin/tazobactam-resistant	*P. aeruginosa*_PiPTaz	Piperacillin, piperacillin/tazobactam
	Multidrug-resistant (MDR)	*P. aeruginosa*_MDR	Cephalosporins, fluoroquinolones, aminoglycosides, carbapenems, piperacillin/tazobactam
*Enterococcus faecium*	Vancomycin-resistant (VRE)	*E. faecium*_VRE	Vancomycin
	Daptomycin-resistant	*E. faecium*_Dapto	Daptomycin (NS)
*Enterococcus faecalis*	Vancomycin-resistant (VRE)	*E. faecalis*_VRE	Vancomycin
	Daptomycin-resistant	*E. faecalis*_Dapto	Daptomycin (NS)
Coagulase-negative *Staphylococci*	Vancomycin-resistant	CNS_Vanc	Vancomycin
Enterobacterales	Carbapenem-resistant (CRE)	CREall	Imipenem, meropenem, doripenem, ertapenem
*Staphylococcus aureus*	Methicillin-resistant (MRSA)	MRSA	Methicillin, oxacillin, cefoxitin
	Linezolid-resistant MRSA	MRSA_Linezolid	Linezolid
	Fluoroquinolone-resistant MRSA	MRSA_Fluoroq	Ciprofloxacin and/or levofloxacin
	Vancomycin-resistant MRSA	MRSA_Vanc	Vancomycin
	Daptomycin-resistant MRSA	MRSA_Dapto	Daptomycin (NS)
*Acinetobacter*	Carbapenem-resistant	*Acinetobacter*_Carbapenems	Imipenem, meropenem, doripenem
	Multidrug-resistant (MDR)	*Acinetobacter*_MDR	Cephalosporins, fluoroquinolones, aminoglycosides, carbapenems, piperacillin/tazobactam, ampicillin/sulbactam

## Data Availability

Not applicable.

## References

[1] Murugaiyan J., Anand Kumar P., Rao G.S., Iskandar K., Hawser S., Hays J.P., Mohsen Y., Adukkadukkam S., Awuah W.A., Jose R.A.M. (2022). Progress in Alternative Strategies to Combat Antimicrobial Resistance: Focus on Antibiotics. Antibiotics.

[2] Rogers J.V., Hall V.L., McOsker C.C. (2022). Crumbling the Castle: Targeting DNABII Proteins for Collapsing Bacterial Biofilms as a Therapeutic Approach to Treat Disease and Combat Antimicrobial Resistance. Antibiotics.

[3] Center for Disease Control (2018). Antibiotic Use in the United States, 2018: Progress and Opportunities.

[4] Ge B., Domesle K.J., Yang Q., Young S.R., Rice-Trujillo C.L., Bodeis Jones S.M., Gaines S.A., Keller M.W., Li X., Piñeiro S.A. (2017). Effects of Low Concentrations of Erythromycin, Penicillin, and Virginiamycin on Bacterial Resistance Development in Vitro. Sci. Rep..

[5] Frost I., Laxminarayan R., McKenna N., Chai S., Joshi J. (2018). Antimicrobial Resistance and Primary Health Care.

[6] Albrich W.C., Monnet D.L., Harbarth S. (2004). Antibiotic Selection Pressure and Resistance in Streptococcus Pneumoniae and Streptococcus Pyogenes. Emerg. Infect. Dis..

[7] Chokshi A., Sifri Z., Cennimo D., Horng H. (2019). Global Contributors to Antibiotic Resistance. J. Glob. Infect. Dis..

[8] Hiller C.X., Hübner U., Fajnorova S., Schwartz T., Drewes J.E. (2019). Antibiotic Microbial Resistance (AMR) Removal Efficiencies by Conventional and Advanced Wastewater Treatment Processes: A Review. Sci. Total Environ..

[9] Fletcher S. (2015). Understanding the Contribution of Environmental Factors in the Spread of Antimicrobial Resistance. Environ. Health Prev. Med..

[10] Savin M., Bierbaum G., Hammerl J.A., Heinemann C., Parcina M., Sib E., Voigt A., Kreyenschmidt J. (2020). Antibiotic-Resistant Bacteria and Antimicrobial Residues in Wastewater and Process Water from German Pig Slaughterhouses and Their Receiving Municipal Wastewater Treatment Plants. Sci. Total Environ..

[11] Danner M.C., Robertson A., Behrends V., Reiss J. (2019). Antibiotic Pollution in Surface Fresh Waters: Occurrence and Effects. Sci. Total Environ..

[12] Petrovich M.L., Zilberman A., Kaplan A., Eliraz G.R., Wang Y., Langenfeld K., Duhaime M., Wigginton K., Poretsky R., Avisar D. (2020). Microbial and Viral Communities and Their Antibiotic Resistance Genes Throughout a Hospital Wastewater Treatment System. Front. Microbiol..

[13] Cabello F.C., Godfrey H.P., Tomova A., Ivanova L., Dölz H., Millanao A., Buschmann A.H. (2013). Antimicrobial Use in Aquaculture Re-Examined: Its Relevance to Antimicrobial Resistance and to Animal and Human Health. Environ. Microbiol..

[14] Shallcross L.J., Howard S.J., Fowler T., Davies S.C. (2015). Tackling the Threat of Antimicrobial Resistance: From Policy to Sustainable Action. Philos. Trans. R. Soc. B Biol. Sci..

[15] Ma Y., Chen J., Fong K., Nadya S., Allen K., Laing C., Ziebell K., Topp E., Carroll L.M., Wiedmann M. (2021). Antibiotic Resistance in Shiga Toxigenic Escherichia Coli Isolates from Surface Waters and Sediments in a Mixed Use Urban Agricultural Landscape. Antibiotics.

[16] Bartley P.S., Domitrovic T.N., Moretto V.T., Santos C.S., Ponce-Terashima R., Reis M.G., Barbosa L.M., Blanton R.E., Bonomo R.A., Perez F. (2019). Antibiotic Resistance in Enterobacteriaceae from Surface Waters in Urban Brazil Highlights the Risks of Poor Sanitation. Am. J. Trop. Med. Hyg..

[17] Santiago-Rodriguez T.M., Rivera J.I., Coradin M., Toranzos G.A. (2013). Antibiotic-Resistance and Virulence Genes in Enterococcus Isolated from Tropical Recreational Waters. J. Water Health.

[18] Silva V., Ferreira E., Manageiro V., Reis L., Tejedor-Junco M.T., Sampaio A., Capelo J.L., Caniça M., Igrejas G., Poeta P. (2021). Distribution and Clonal Diversity of Staphylococcus Aureus and Other Staphylococci in Surface Waters: Detection of ST425-T742 and ST130-T843 MecC-Positive MRSA Strains. Antibiotics.

[19] Hay S.I., Rao P.C., Dolecek C., Day N.P.J., Stergachis A., Lopez A.D., Murray C.J.L. (2018). Measuring and Mapping the Global Burden of Antimicrobial Resistance. BMC Med..

[20] Samreen, Ahmad I., Malak H.A., Abulreesh H.H. (2021). Environmental Antimicrobial Resistance and Its Drivers: A Potential Threat to Public Health. J. Glob. Antimicrob. Resist..

[21] Raffatellu M. (2018). Learning from Bacterial Competition in the Host to Develop Antimicrobials. Nat. Med..

[22] McInnes R.S., uz-Zaman M.H., Alam I.T., Ho S.F.S., Moran R.A., Clemens J.D., Islam M.S., van Schaik W. (2021). Metagenome-Wide Analysis of Rural and Urban Surface Waters and Sediments in Bangladesh Identifies Human Waste as a Driver of Antibiotic Resistance. mSystems.

[23] Zhang C.M., Liang J., Liu W.Y. (2021). Comparative Study on the Bacterial Diversity and Antibiotic Resistance Genes of Urban Landscape Waters Replenished by Reclaimed Water and Surface Water in Xi’an, China. Environ. Sci. Pollut. Res..

[24] Zhang S.H., Lv X., Han B., Gu X., Wang P.F., Wang C., He Z. (2015). Prevalence of Antibiotic Resistance Genes in Antibiotic-Resistant Escherichia Coli Isolates in Surface Water of Taihu Lake Basin, China. Environ. Sci. Pollut. Res..

[25] Liang X., Guan F., Chen B., Luo P., Guo C., Wu G., Ye Y., Zhou Q., Fang H. (2020). Spatial and Seasonal Variations of Antibiotic Resistance Genes and Antibiotics in the Surface Waters of Poyang Lake in China. Ecotoxicol. Environ. Saf..

[26] Zhao B., Xu J., Zhang G., Lu S., Liu X., Li L., Li M. (2021). Occurrence of Antibiotics and Antibiotic Resistance Genes in the Fuxian Lake and Antibiotic Source Analysis Based on Principal Component Analysis-Multiple Linear Regression Model. Chemosphere.

[27] Zhang S., Pang S., Wang P.F., Wang C., Han N., Liu B., Han B., Li Y., Anim-Larbi K. (2016). Antibiotic Concentration and Antibiotic-Resistant Bacteria in Two Shallow Urban Lakes after Stormwater Event. Environ. Sci. Pollut. Res..

[28] Wang Z., Han M., Li E., Liu X., Wei H., Yang C., Lu S., Ning K. (2020). Distribution of Antibiotic Resistance Genes in an Agriculturally Disturbed Lake in China: Their Links with Microbial Communities, Antibiotics, and Water Quality. J. Hazard. Mater..

[29] Yang Y., Xu C., Cao X., Lin H., Wang J. (2017). Antibiotic Resistance Genes in Surface Water of Eutrophic Urban Lakes Are Related to Heavy Metals, Antibiotics, Lake Morphology and Anthropic Impact. Ecotoxicology.

[30] Wang C., Gu X., Zhang S., Wang P., Guo C., Gu J., Hou J. (2013). Characterization of Antibiotic-Resistance Genes in Antibiotic Resistance Escherichia Coli Isolates from a Lake. Arch. Environ. Contam. Toxicol..

[31] Moussa J., Abboud E., Tokajian S. (2021). The Dissemination of Antimicrobial Resistance Determinants in Surface Water Sources in Lebanon. FEMS Microbiol. Ecol..

[32] Reichert G., Hilgert S., Alexander J., Rodrigues de Azevedo J.C., Morck T., Fuchs S., Schwartz T. (2021). Determination of Antibiotic Resistance Genes in a WWTP-Impacted River in Surface Water, Sediment, and Biofilm: Influence of Seasonality and Water Quality. Sci. Total Environ..

[33] Cheng J., Tang X., Liu C. (2020). Occurrence and Distribution of Antibiotic Resistance Genes in Various Rural Environmental Media. Environ. Sci. Pollut. Res..

[34] Wu D.L., Zhang M., He L.X., Zou H.Y., Liu Y.S., Li B.B., Yang Y.Y., Liu C., He L.Y., Ying G.G. (2020). Contamination Profile of Antibiotic Resistance Genes in Ground Water in Comparison with Surface Water. Sci. Total Environ..

[35] Stange C., Yin D., Xu T., Guo X., Schäfer C., Tiehm A. (2019). Distribution of Clinically Relevant Antibiotic Resistance Genes in Lake Tai, China. Sci. Total Environ..

[36] Jiang H., Zhou R., Yang Y., Chen B., Cheng Z., Zhang M., Li J., Zhang G., Zou S. (2018). Characterizing the Antibiotic Resistance Genes in a River Catchment: Influence of Anthropogenic Activities. J. Environ. Sci..

[37] Jia J., Gomes-Silva G., Plath M., Pereira B.B., UeiraVieira C., Wang Z. (2021). Shifts in Bacterial Communities and Antibiotic Resistance Genes in Surface Water and Gut Microbiota of Guppies (Poecilia Reticulata) in the Upper Rio Uberabinha, Brazil. Ecotoxicol. Environ. Saf..

[38] Arsand J.B., Hoff R.B., Jank L., Bussamara R., Dallegrave A., Bento F.M., Kmetzsch L., Falção D.A., do Carmo Ruaro Peralba M., de Araujo Gomes A. (2020). Presence of Antibiotic Resistance Genes and Its Association with Antibiotic Occurrence in Dilúvio River in Southern Brazil. Sci. Total Environ..

[39] Garner E., Benitez R., von Wagoner E., Sawyer R., Schaberg E., Hession W.C., Krometis L.A.H., Badgley B.D., Pruden A. (2017). Stormwater Loadings of Antibiotic Resistance Genes in an Urban Stream. Water Res..

[40] Liyanage G.Y., Illango A., Manage P.M. (2021). Prevalence and Quantitative Analysis of Antibiotic Resistance Genes (ARGs) in Surface and Groundwater in Meandering Part of the Kelani River Basin in Sri Lanka. Water. Air. Soil Pollut..

[41] Jiang X., Liu L., Chen J., Fan X., Xie S., Huang J., Yu G. (2021). Antibiotic Resistance Genes and Mobile Genetic Elements in a Rural River in Southeast China: Occurrence, Seasonal Variation and Association with the Antibiotics. Sci. Total Environ..

[42] Tang Y., Liang Z., Li G., Zhao H., An T. (2021). Metagenomic Profiles and Health Risks of Pathogens and Antibiotic Resistance Genes in Various Industrial Wastewaters and the Associated Receiving Surface Water. Chemosphere.

[43] Chaturvedi P., Chowdhary P., Singh A., Chaurasia D., Pandey A., Chandra R., Gupta P. (2021). Dissemination of Antibiotic Resistance Genes, Mobile Genetic Elements, and Efflux Genes in Anthropogenically Impacted Riverine Environments. Chemosphere.

[44] Stange C., Sidhu J.P.S., Tiehm A., Toze S. (2016). Antibiotic Resistance and Virulence Genes in Coliform Water Isolates. Int. J. Hyg. Environ. Health.

[45] Guruge K.S., Tamamura Y.A., Goswami P., Tanoue R., Jinadasa K.B.S.N., Nomiyama K., Ohura T., Kunisue T., Tanabe S., Akiba M. (2021). The Association between Antimicrobials and the Antimicrobial-Resistant Phenotypes and Resistance Genes of Escherichia Coli Isolated from Hospital Wastewaters and Adjacent Surface Waters in Sri Lanka. Chemosphere.

[46] Molale L.G., Bezuidenhout C.C. (2016). Antibiotic Resistance, Efflux Pump Genes and Virulence Determinants in Enterococcus Spp. from Surface Water Systems. Environ. Sci. Pollut. Res..

[47] Kaushik M., Khare N., Kumar S., Gulati P. (2019). High Prevalence of Antibiotic Resistance and Integrons in Escherichia Coli Isolated from Urban River Water, India. Microb. Drug Resist..

[48] Azevedo J.S.N., Araújo S., Oliveira C.S., Correia A., Henriques I. (2013). Analysis of Antibiotic Resistance in Bacteria Isolated from the Surface Microlayer and Underlying Water of an Estuarine Environment. Microb. Drug Resist..

[49] Veljović K., Popović N., Vidojević A.T., Tolinački M., Mihajlović S., Jovčić B., Kojić M. (2015). Environmental Waters as a Source of Antibiotic-Resistant Enterococcus Species in Belgrade, Serbia. Environ. Monit. Assess..

[50] Falgenhauer L., Schwengers O., Schmiedel J., Baars C., Lambrecht O., Heß S., Berendonk T.U., Falgenhauer J., Chakraborty T., Imirzalioglu C. (2019). Multidrug-Resistant and Clinically Relevant Gram-Negative Bacteria Are Present in German Surface Waters. Front. Microbiol..

[51] Stoll C., Sidhu J.P.S., Tiehm A., Toze S. (2012). Prevalence of Clinically Relevant Antibiotic Resistance Genes in Surface Water Samples Collected from Germany and Australia. Environ. Sci. Technol..

[52] Kotwani A., Joshi J., Kaloni D. (2021). Pharmaceutical Effluent: A Critical Link in the Interconnected Ecosystem Promoting Antimicrobial Resistance. Environ. Sci. Pollut. Res..

[53] Larsson D.G.J. (2014). Pollution from Drug Manufacturing: Review and Perspectives. Philos. Trans. R. Soc. B Biol. Sci..

[54] Bengtsson-Palme J., Larsson D.G.J. (2016). Concentrations of Antibiotics Predicted to Select for Resistant Bacteria: Proposed Limits for Environmental Regulation. Environ. Int..

[55] Arya G., Tadayon S., Sadighian J., Jones J., de Mutsert K., Huff T.B., Foster G. (2017). Pharmaceutical Chemicals, Steroids and Xenoestrogens in Water, Sediments and Fish from the Tidal Freshwater Potomac River (Virginia, USA). J. Environ. Sci. Health Part A.

[56] Burke V., Richter D., Greskowiak J., Mehrtens A., Schulz L., Massmann G. (2016). Occurrence of Antibiotics in Surface and Groundwater of a Drinking Water Catchment Area in Germany. Water Environ. Res..

[57] Dodgen L.K., Kelly W.R., Panno S.V., Taylor S.J., Armstrong D.L., Wiles K.N., Zhang Y., Zheng W. (2017). Characterizing Pharmaceutical, Personal Care Product, and Hormone Contamination in a Karst Aquifer of Southwestern Illinois, USA, Using Water Quality and Stream Flow Parameters. Sci. Total Environ..

[58] Jurado A., Walther M., Díaz-Cruz M.S. (2019). Occurrence, Fate and Environmental Risk Assessment of the Organic Microcontaminants Included in the Watch Lists Set by EU Decisions 2015/495 and 2018/840 in the Groundwater of Spain. Sci. Total Environ..

[59] Centers for Disease Control and Prevention (CDC) Antibiotic Resistance & Patient Safety Portal. https://arpsp.cdc.gov/about?tab=antibiotic-resistance.

[60] Andersson D.I., Hughes D. (2014). Microbiological Effects of Sublethal Levels of Antibiotics. Nat. Rev. Microbiol..

[61] Rozman U., Duh D., Cimerman M., Turk S.Š. (2020). Hospital Wastewater Effluent: Hot Spot for Antibiotic Resistant Bacteria. J. Water Sanit. Hyg. Dev..

[62] Velpandian T., Halder N., Nath M., Das U., Moksha L., Gowtham L., Batta S.P. (2018). Un-Segregated Waste Disposal: An Alarming Threat of Antimicrobials in Surface and Ground Water Sources in Delhi. Environ. Sci. Pollut. Res..

[63] Han S., Li X., Huang H., Wang T., Wang Z., Fu X., Zhou Z., Du P., Li X. (2021). Simultaneous Determination of Seven Antibiotics and Five of Their Metabolites in Municipal Wastewater and Evaluation of Their Stability under Laboratory Conditions. Int. J. Environ. Res. Public Health.

[64] Cars O., Nordberg P. (2005). Antibiotic Resistance—The Faceless Threat. Int. J. Risk Saf. Med..

[65] Morgan D.J., Okeke I.N., Laxminarayan R., Perencevich E.N., Weisenberg S. (2011). Non-Prescription Antimicrobial Use Worldwide: A Systematic Review. Lancet Infect. Dis..

[66] Esimone C.O., Nworu C.S., Udeogaranya O.P. (2007). Utilization of Antimicrobial Agents with and without Prescription by Out-Patients in Selected Pharmacies in South-Eastern Nigeria. Pharm. World Sci..

[67] World Health Organization (2009). Community-Based Surveillance of Antimicrobial Use and Resistance in Resource-Constrained Settings Report on Five Pilot Projects.

[68] Alam M.U., Rahman M., Abdullah-Al-Masud, Islam M.A., Asaduzzaman M., Sarker S., Rousham E., Unicomb L. (2019). Human Exposure to Antimicrobial Resistance from Poultry Production: Assessing Hygiene and Waste-Disposal Practices in Bangladesh. Int. J. Hyg. Environ. Health.

[69] Homeier-Bachmann T., Heiden S.E., Lübcke P.K., Bachmann L., Bohnert J.A., Zimmermann D., Schaufler K. (2021). Antibiotic-Resistant Enterobacteriaceae in Wastewater of Abattoirs. Antibiotics.

[70] Savin M., Bierbaum G., Kreyenschmidt J., Schmithausen R.M., Sib E., Schmoger S., Käsbohrer A., Hammerl J.A. (2021). Clinically Relevant Escherichia Coli Isolates from Process Waters and Wastewater of Poultry and Pig Slaughterhouses in Germany. Microorganisms.

[71] EPA (2002). Wastewater Technology Fact Sheet—Disinfection for Small Systems.

[72] Łuczkiewicz A., Jankowska K., Fudala-Ksiazek S., Olańczuk-Neyman K. (2010). Antimicrobial Resistance of Fecal Indicators in Municipal Wastewater Treatment Plant. Water Res..

[73] Haywood J., Vadlamani G., Stubbs K.A., Mylne J.S. (2021). Antibiotic Resistance Lessons for the Herbicide Resistance Crisis. Pest Manag. Sci..

[74] Van Hoek A.H.A.M., Mevius D., Guerra B., Mullany P., Roberts A.P., Aarts H.J.M. (2011). Acquired Antibiotic Resistance Genes: An Overview. Front. Microbiol..

[75] Mazel D., Davies J. (1999). Antibiotic Resistance in Microbes. Cell. Mol. Life Sci..

[76] Novo A., André S., Viana P., Nunes O.C., Manaia C.M. (2013). Antibiotic Resistance, Antimicrobial Residues and Bacterial Community Composition in Urban Wastewater. Water Res..

[77] Wei R., Ge F., Huang S., Chen M., Wang R. (2011). Occurrence of Veterinary Antibiotics in Animal Wastewater and Surface Water around Farms in Jiangsu Province, China. Chemosphere.

[78] Gotkowska-Płachta A. (2021). The Prevalence of Virulent and Multidrug-Resistant Enterococci in River Water and in Treated and Untreated Municipal and Hospital Wastewater. Int. J. Environ. Res. Public Health.

[79] Zhang Y., Marrs C.F., Simon C., Xi C. (2009). Wastewater Treatment Contributes to Selective Increase of Antibiotic Resistance among Acinetobacter Spp.. Sci. Total Environ..

[80] Agga G.E., Kasumba J., Loughrin J.H., Conte E.D. (2020). Anaerobic Digestion of Tetracycline Spiked Livestock Manure and Poultry Litter Increased the Abundances of Antibiotic and Heavy Metal Resistance Genes. Front. Microbiol..

[81] Ceccarelli D., Hesp A., Van Der Goot J., Joosten P., Sarrazin S., Wagenaar J.A., Dewulf J., Mevius D.J. (2020). Antimicrobial Resistance Prevalence in Commensal Escherichia Coli from Broilers, Fattening Turkeys, Fattening Pigs and Veal Calves in European Countries and Association with Antimicrobial Usage at Country Level. J. Med. Microbiol..

[82] Innes G.K., Randad P.R., Korinek A., Davis M.F., Price L.B., So A.D., Heaney C.D. (2019). External Societal Costs of Antimicrobial Resistance in Humans Attributable to Antimicrobial Use in Livestock. Annu. Rev. Public Health.

[83] Lhermie G., Tauer L.W., Gröhn Y.T. (2018). An Assessment of the Economic Costs to the U.S. Dairy Market of Antimicrobial Use Restrictions. Prev. Vet. Med..

[84] Boxall A.B.A., Hardy A., Beulke S., Boucard T., Burgin L., Falloon P.D., Haygarth P.M., Hutchinson T., Kovats R.S., Leonardi G. (2009). Impacts of Climate Change on Indirect Human Exposure to Pathogens and Chemicals from Agriculture. Environ. Health Perspect..

[85] Hall M.C., Duerschner J., Gilley J.E., Schmidt A.M., Bartelt-Hunt S.L., Snow D.D., Eskridge K.M., Li X. (2021). Antibiotic Resistance Genes in Swine Manure Slurry as Affected by Pit Additives and Facility Disinfectants. Sci. Total Environ..

[86] MacDonald E., White R., Mexia R., Bruun T., Kapperud G., Lange H., Nygard K., Vold L. (2015). Risk Factors for Sporadic Domestically Acquired Campylobacter Infections in Norway 2010-2011: A National Prospective Case-Control Study. PLoS ONE.

[87] Verraes C., Van Boxstael S., Van Meervenne E., Van Coillie E., Butaye P., Catry B., de Schaetzen M.A., Van Huffel X., Imberechts H., Dierick K. (2013). Antimicrobial Resistance in the Food Chain: A Review. Int. J. Environ. Res. Public Health.

[88] Morrison L., Zembower T.R. (2020). Antimicrobial Resistance. Gastrointest. Endosc. Clin. N. Am..

[89] Hölzel C.S., Tetens J.L., Schwaiger K. (2018). Unraveling the Role of Vegetables in Spreading Antimicrobial-Resistant Bacteria: A Need for Quantitative Risk Assessment. Foodborne Pathog. Dis..

[90] Ulger T.G., Songur A.N., Cirak O., Cakiroglu F. (2018). Role of Vegetable in Human Nutrition and Disease Prevention.

[91] van Hoek A.H.A.M., Veenman C., van Overbeek W.M., Lynch G., de Roda Husman A.M., Blaak H. (2015). Prevalence and Characterization of ESBL- and AmpC-Producing Enterobacteriaceae on Retail Vegetables. Int. J. Food Microbiol..

[92] Chee-Sanford J.C., Mackie R.I., Koike S., Krapac I.G., Lin Y.-F., Yannarell A.C., Maxwell S., Aminov R.I. (2009). Fate and Transport of Antibiotic Residues and Antibiotic Resistance Genes Following Land Application of Manure Waste. J. Environ. Qual..

[93] Jung Y., Jang H., Matthews K.R. (2014). Effect of the Food Production Chain from Farm Practices to Vegetable Processing on Outbreak Incidence. Microb. Biotechnol..

[94] He Y., Yuan Q., Mathieu J., Stadler L., Senehi N., Sun R., Alvarez P.J.J. (2020). Antibiotic Resistance Genes from Livestock Waste: Occurrence, Dissemination, and Treatment. NPJ Clean Water.

[95] Centers for Disease Control and Prevention (CDC) Where Resistance Spreads: Food Supply Medical Professional with Young Female Patient. https://www.cdc.gov/drugresistance/food.html.

[96] Centers for Disease Control and Prevention (CDC) Outbreak of Multidrug-Resistant Salmonella Infections Linked to Raw Chicken Products. https://www.cdc.gov/salmonella/infantis-10-18/index.html.

[97] Van Boxstael S., Dierick K., Van Huffel X., Uyttendaele M., Berkvens D., Herman L., Bertrand S., Wildemauwe C., Catry B., Butaye P. (2012). Comparison of Antimicrobial Resistance Patterns and Phage Types of Salmonella Typhimurium Isolated from Pigs, Pork and Humans in Belgium between 2001 and 2006. Food Res. Int..

[98] Walker R.A., Lawson A.J., Lindsay E.A., Ward L.R., Wright P.A., Bolton F.J., Wareing D.R.A., Corkish J.D., Davies R.H., Threlfall E.J. (2000). Decreased Susceptibility to Ciprofloxacin in Outbreak-Associated Multiresistant Salmonella Typhimurium DT104. Vet. Rec..

[99] Cody S.H., Abbott S.L., Marfin A.A., Schulz B., Wagner P., Robbins K., Mohle-Boetani J.C., Vugia D.J. (1999). Two Outbreaks of Multidrug-Resistant Salmonella Serotype Typhimurium DT104 Infections Linked to Raw-Milk Cheese in Northern California. J. Am. Med. Assoc..

[100] Ling M.L., Goh K.T., Wang G.C.Y., Neo K.S., Chua T. (2002). An Outbreak of Multidrug-Resistant Salmonella Enterica Subsp. Enterica Serotype Typhimurium, DT104L Linked to Dried Anchovy in Singapore. Epidemiol. Infect..

[101] Horby P.W., O’Brien S.J., Adak G.K., Graham C., Hawker J.I., Hunter P., Lane C., Lawson A.J., Mitchell R.T., Reacher M.H. (2003). A National Outbreak of Multi-Resistant Salmonella Enterica Serovar Typhimurium Definitive Phage Type (DT) 104 Associated with Consumption of Lettuce. Epidemiol. Infect..

[102] Ethelberg S., Sørensen G., Kristensen B., Christensen K., Krusell L., Hempel-Jørgensen A., Perge A., Nielsen E.M. (2007). Outbreak with Multi-Resistant Salmonella Typhimurium DT104 Linked to Carpaccio, Denmark, 2005. Epidemiol. Infect..

[103] Ellis-Iversen J., Seyfarth A.M., Korsgaard H., Bortolaia V., Munck N., Dalsgaard A. (2020). Antimicrobial Resistant, E. Coli and Enterococci in Pangasius Fillets and Prawns in Danish Retail Imported from Asia. Food Control.

[104] Ferreira A.C.A.D.O., Pavelquesi S.L.S., Monteiro E.D.S., Rodrigues L.F.S., Silva C.M.D.S., Da Silva I.C.R., Orsi D.C. (2021). Prevalence and Antimicrobial Resistance of Salmonella Spp. In Aquacultured Nile Tilapia (Oreochromis Niloticus) Commercialized in Federal District, Brazil. Foodborne Pathog. Dis..

[105] Teuber M., Meile L., Schwartz F. (1999). Acquired Antibiotic Resistance in Lactic Acid Bacteria from Foods. Antonie Van Leeuwenhoek.

[106] Masco L., Van Hoorde K., De Brandt E., Swings J., Huys G. (2006). Antimicrobial Susceptibility of Bifidobacterium Strains from Humans, Animals and Probiotic Products. J. Antimicrob. Chemother..

[107] Gevers D., Masco L., Baert L., Huys G., Debevere J., Swings J. (2003). Prevalence and Diversity of Tetracycline Resistant Lactic Acid Bacteria and Their Tet Genes along the Process Line of Fermented Dry Sausages. Syst. Appl. Microbiol..

[108] Kastner S., Perreten V., Bleuler H., Hugenschmidt G., Lacroix C., Meile L. (2006). Antibiotic Susceptibility Patterns and Resistance Genes of Starter Cultures and Probiotic Bacteria Used in Food. Syst. Appl. Microbiol..

[109] Resch M., Nagel V., Hertel C. (2008). Antibiotic Resistance of Coagulase-Negative Staphylococci Associated with Food and Used in Starter Cultures. Int. J. Food Microbiol..

[110] Smith T.C., Gebreyes W.A., Abley M.J., Harper A.L., Forshey B.M., Male M.J., Martin H.W., Molla B.Z., Sreevatsan S., Thakur S. (2013). Methicillin-Resistant Staphylococcus Aureus in Pigs and Farm Workers on Conventional and Antibiotic-Free Swine Farms in the USA. PLoS ONE.

[111] Pérez-Rodríguez F., Mercanoglu Taban B. (2019). A State-of-Art Review on Multi-Drug Resistant Pathogens in Foods of Animal Origin: Risk Factors and Mitigation Strategies. Front. Microbiol..

[112] Sharma D., Sharma P.K., Malik A. (2011). Prevalence and Antimicrobial Susceptibility of Drug Resistant Staphylococcus Aureus in Raw Milk of Dairy Cattle. Int. Res. J. Microbiol..

[113] Woolhouse M.E.J., Ward M.J. (2013). Sources of Antimicrobial Resistance. Science.

[114] Alcaine S.D., Warnick L.D., Wiedmann M. (2007). Antimicrobial Resistance in Nontyphoidal Salmonella. J. Food Prot..

[115] Roca I., Akova M., Baquero F., Carlet J., Cavaleri M., Coenen S., Cohen J., Findlay D., Gyssens I., Heure O.E. (2015). The Global Threat of Antimicrobial Resistance: Science for Intervention. New Microbes New Infect..

[116] Wang X., Biswas S., Paudyal N., Pan H., Li X., Fang W., Yue M. (2019). Antibiotic Resistance in Salmonella Typhimurium Isolates Recovered from the Food Chain through National Antimicrobial Resistance Monitoring System between 1996 and 2016. Front. Microbiol..

[117] Oniciuc E.A., Likotrafiti E., Alvarez-Molina A., Prieto M., López M., Alvarez-Ordóñez A. (2019). Food Processing as a Risk Factor for Antimicrobial Resistance Spread along the Food Chain. Curr. Opin. Food Sci..

[118] Barber D.A., Miller G.Y., McNamara P.E. (2003). Models of Antimicrobial Resistance and Foodborne Illness: Examining Assumptions and Practical Applications. J. Food Prot..

[119] Ou C., Shang D., Yang J., Chen B., Chang J., Jin F., Shi C. (2020). Prevalence of Multidrug-Resistant Staphylococcus Aureus Isolates with Strong Biofilm Formation Ability among Animal-Based Food in Shanghai. Food Control.

[120] Bengtsson-Palme J., Kristiansson E., Larsson D.G.J. (2018). Environmental Factors Influencing the Development and Spread of Antibiotic Resistance. FEMS Microbiol. Rev..

[121] Swift B.M.C., Bennett M., Waller K., Dodd C., Murray A., Gomes R.L., Humphreys B., Hobman J.L., Jones M.A., Whitlock S.E. (2019). Anthropogenic Environmental Drivers of Antimicrobial Resistance in Wildlife. Sci. Total Environ..

[122] Carroll D., Wang J., Fanning S., Mcmahon B.J. (2015). Antimicrobial Resistance in Wildlife: Implications for Public Health. Zoonoses Public Health.

[123] Allen H.K., Donato J., Wang H.H., Cloud-Hansen K.A., Davies J., Handelsman J. (2010). Call of the Wild: Antibiotic Resistance Genes in Natural Environments. Nat. Rev. Microbiol..

[124] Yuan Y., Liang B., Jiang B.W., Zhu L.W., Wang T.C., Li Y.G., Liu J., Guo X.J., Ji X., Sun Y. (2021). Migratory Wild Birds Carrying Multidrug-Resistant Escherichia Coli as Potential Transmitters of Antimicrobial Resistance in China. PLoS ONE.

[125] Viceconte G., Petrosillo N. (2020). COVID-19 R0: Magic Number or Conundrum?. Infect. Dis. Rep..

[126] Malvy D., McElroy A.K., de Clerck H., Günther S., van Griensven J. (2019). Ebola Virus Disease. Lancet.

[127] Qiu Z., Giri S., Wang L., Luo B. (2018). SWAT Modeling of Fecal Indicator Bacteria Fate and Transport in a Suburban Watershed with Mixed Land Uses. Proc. Int. Acad. Ecol. Environ. Sci..

[128] Rolland R.M., Hausfater G., Marshall B., Levy S.B. (1985). Antibiotic-Resistant Bacteria in Wild Primates: Increased Prevalence in Baboons Feeding on Human Refuse. Appl. Environ. Microbiol..

[129] Rwego I.B., Isabirye-Basuta G., Gillespie T.R., Goldberg T.L. (2008). Gastrointestinal Bacterial Transmission among Humans, Mountain Gorillas, and Livestock in Bwindi Impenetrable National Park, Uganda. Conserv. Biol..

[130] Bonnedahl J., Drobni M., Gauthier-Clerc M., Hernandez J., Granholm S., Kayser Y., Melhus Å., Kahlmeter G., Waldenström J., Johansson A. (2009). Dissemination of Escherichia Coli with CTX-M Type ESBL between Humans and Yellow-Legged Gulls in the South of France. PLoS ONE.

[131] Thaller M.C., Migliore L., Marquez C., Tapia W., Cedeño V., Rossolini G.M., Gentile G. (2010). Tracking Acquired Antibiotic Resistance in Commensal Bacteria of Galápagos Land Iguanas: No Man, No Resistance. PLoS ONE.

[132] Van Goethem M.W., Pierneef R., Bezuidt O.K.I., Van De Peer Y., Cowan D.A., Makhalanyane T.P. (2018). A Reservoir of ‘Historical’ Antibiotic Resistance Genes in Remote Pristine Antarctic Soils. Microbiome.

[133] Tenover F.C. (2006). Mechanisms of Antimicrobial Resistance in Bacteria. Am. J. Med..

[134] Fountain J.C., Koh J., Yang L., Pandey M.K., Nayak S.N., Bajaj P., Zhuang W.J., Chen Z.Y., Kemerait R.C., Lee R.D. (2018). Proteome Analysis of Aspergillus Flavus Isolate-Specific Responses to Oxidative Stress in Relationship to Aflatoxin Production Capability. Sci. Rep..

[135] McCormick A., Whitney C., Farley M., Lynfield R., Harrison L.H., Bennett N.M., Schaffner W., Reingold A., Hadler J., Cieslak P. (2003). Geographic Diversity and Temporal Trends of Antimicrobial Resistance in Streptococcus Pneumoniae in the United States. Nat. Med..

[136] Nicholson F.A., Smith S.R., Alloway B.J., Carlton-Smith C., Chambers B.J. (2003). An Inventory of Heavy Metals Inputs to Agricultural Soils in England and Wales. Sci. Total Environ..

[137] Hadi E.H. (2015). Characterization of Soil Heavy Metal Contamination in the Abandoned Mine of Zaida (High Moulouya, Morocco). Int. Res. J. Earth Sci..

[138] Hussey S.J.K., Purves J., Allcock N., Fernandes V.E., Monks P.S., Ketley J.M., Andrew P.W., Morrissey J.A. (2017). Air Pollution Alters Staphylococcus Aureus and Streptococcus Pneumoniae Biofilms, Antibiotic Tolerance and Colonisation. Environ. Microbiol..

[139] Gao P., He S., Huang S., Li K., Liu Z., Xue G., Sun W. (2015). Impacts of Coexisting Antibiotics, Antibacterial Residues, and Heavy Metals on the Occurrence of Erythromycin Resistance Genes in Urban Wastewater. Appl. Microbiol. Biotechnol..

[140] Wright M.S., Peltier G.L., Stepanauskas R., McArthur J.V. (2006). Bacterial Tolerances to Metals and Antibiotics in Metal-Contaminated and Reference Streams. FEMS Microbiol. Ecol..

[141] Berg J., Thorsen M.K., Holm P.E., Jensen J., Nybroe O., Brandt K.K. (2010). Cu Exposure under Field Conditions Coselects for Antibiotic Resistance as Determined by a Novel Cultivation-Independent Bacterial Community Tolerance Assay. Environ. Sci. Technol..

[142] Chen J., Li J., Zhang H., Shi W., Liu Y. (2019). Bacterial Heavy-Metal and Antibiotic Resistance Genes in a Copper Tailing Dam Area in Northern China. Front. Microbiol..

[143] Azarbad H., Niklińska M., Laskowski R., van Straalen N.M., van Gestel C.A.M., Zhou J., He Z., Wen C., Röling W.F.M. (2015). Microbial Community Composition and Functions Are Resilient to Metal Pollution along Two Forest Soil Gradients. FEMS Microbiol. Ecol..

[144] Dickinson A.W., Power A., Hansen M.G., Brandt K.K., Piliposian G., Appleby P., O’Neill P.A., Jones R.T., Sierocinski P., Koskella B. (2019). Heavy Metal Pollution and Co-Selection for Antibiotic Resistance: A Microbial Palaeontology Approach. Environ. Int..

[145] Chen Y., Jiang Y., Huang H., Mou L., Ru J., Zhao J., Xiao S. (2018). Long-Term and High-Concentration Heavy-Metal Contamination Strongly Influences the Microbiome and Functional Genes in Yellow River Sediments. Sci. Total Environ..

[146] Zhang M., Chen L., Ye C., Yu X. (2018). Co-Selection of Antibiotic Resistance via Copper Shock Loading on Bacteria from a Drinking Water Bio-Filter. Environ. Pollut..

[147] Shi P., Jia S., Zhang X.X., Zhang T., Cheng S., Li A. (2013). Metagenomic Insights into Chlorination Effects on Microbial Antibiotic Resistance in Drinking Water. Water Res..

[148] Baker-Austin C., Wright M.S., Stepanauskas R., McArthur J.V. (2006). Co-Selection of Antibiotic and Metal Resistance. Trends Microbiol..

[149] Imran M., Das K.R., Naik M.M. (2019). Co-Selection of Multi-Antibiotic Resistance in Bacterial Pathogens in Metal and Microplastic Contaminated Environments: An Emerging Health Threat. Chemosphere.

[150] Zou H., He L., Gao F., Zhang M., Chen S., Wu D., Liu Y., He L., Bai H., Ying G. (2021). Antibiotic Resistance Genes in Surface Water and Groundwater from Mining Affected Environments. Sci. Total Environ..

[151] Rizzo L., Manaia C., Merlin C., Schwartz T., Dagot C., Ploy M.C., Michael I., Fatta-Kassinos D. (2013). Urban Wastewater Treatment Plants as Hotspots for Antibiotic Resistant Bacteria and Genes Spread into the Environment: A Review. Sci. Total Environ..

[152] Stepanauskas R., Glenn T.C., Jagoe C.H., Tuckfield R.C., Lindell A.H., McArthur J.V. (2005). Elevated Microbial Tolerance to Metals and Antibiotics in Metal-Contaminated Industrial Environments. Environ. Sci. Technol..

[153] Chen S., Li X., Sun G., Zhang Y., Su J., Ye J. (2015). Heavy Metal Induced Antibiotic Resistance in Bacterium LSJC7. Int. J. Mol. Sci..

[154] Kusi J., Scheuerman P.R., Maier K.J. (2020). Antimicrobial Properties of Silver Nanoparticles May Interfere with Fecal Indicator Bacteria Detection in Pathogen Impaired Streams. Environ. Pollut..

[155] Kusi J., Scheuerman P.R., Maier K.J. (2020). Emerging Environmental Contaminants (Silver Nanoparticles) Altered the Catabolic Capability and Metabolic Fingerprinting of Microbial Communities. Aquat. Toxicol..

[156] Liao S., Zhang Y., Pan X., Zhu F., Jiang C., Liu Q., Cheng Z., Dai G., Wu G., Wang L. (2019). Antibacterial Activity and Mechanism of Silver Nanoparticles against Multidrug-Resistant Pseudomonas Aeruginosa. Int. J. Nanomed..

[157] Mann R., Holmes A., McNeilly O., Cavaliere R., Sotiriou G.A., Rice S.A., Gunawan C. (2021). Evolution of Biofilm-Forming Pathogenic Bacteria in the Presence of Nanoparticles and Antibiotic: Adaptation Phenomena and Cross-Resistance. J. Nanobiotechnol..

[158] Lu J., Wang Y., Jin M., Yuan Z., Bond P., Guo J. (2020). Both Silver Ions and Silver Nanoparticles Facilitate the Horizontal Transfer of Plasmid-Mediated Antibiotic Resistance Genes. Water Res..

[159] Stabryla L.M., Johnston K.A., Diemler N.A., Cooper V.S., Millstone J.E., Haig S.J., Gilbertson L.M. (2021). Role of Bacterial Motility in Differential Resistance Mechanisms of Silver Nanoparticles and Silver Ions. Nat. Nanotechnol..

[160] Skaland R.G., Herrador B.G., Hisdal H., Hygen H.O., Hyllestad S., Lund V., White R., Wong W.K., Nygård K. (2022). Impacts of Climate Change on Drinking Water Quality in Norway. J. Water Health.

[161] Okaka F.O., Odhiambo B.D.O. (2018). Relationship between Flooding and out Break of Infectious Diseasesin Kenya: A Review of the Literature. J. Environ. Public Health.

[162] Saulnier D.D., Hanson C., Ir P., Alvesson H.M., von Schreeb J. (2018). The Effect of Seasonal Floods on Health: Analysis of Six Years of National Health Data and Flood Maps. Int. J. Environ. Res. Public Health.

[163] Williamson S. (2008). Economic Impacts of Climate Change on Colorado Contributors to the Report.

[164] Burnham J.P. (2021). Climate Change and Antibiotic Resistance: A Deadly Combination. Ther. Adv. Infect. Dis..

[165] MacFadden D.R., McGough S.F., Fisman D., Santillana M., Brownstein J.S. (2018). Antibiotic Resistance Increases with Local Temperature. Nat. Clim. Chang..

[166] Rodrigues D.F., Jaisi D.P., Elimelech M. (2013). Toxicity of Functionalized Single-Walled Carbon Nanotubes on Soil Microbial Communities: Implications for Nutrient Cycling in Soil. Environ. Sci. Technol..

[167] Patz J., Githeko A., McCarty J. (2003). Chapter 6: Climate Change and Infectious Diseases. Climate Change and Human Health: Risks and Responses.

[168] Treacy J., Potgieter N., Traore A.N. (2019). Drinking Water Treatment and Challenges in Developing Countries. The Relevance of Hygiene to Health in Developing Countries.

